# The Influence of Groundwater Depletion from Irrigated Agriculture on the Tradeoffs between Ecosystem Services and Economic Returns

**DOI:** 10.1371/journal.pone.0168681

**Published:** 2016-12-29

**Authors:** Kent Kovacs, Grant West

**Affiliations:** Department of Agricultural Economics and Agribusiness, University of Arkansas, Fayetteville, Arkansas, United States of America; University of Vermont, UNITED STATES

## Abstract

An irrigated agricultural landscape experiencing groundwater overdraft generates economic returns and a suite of ecosystem services (in particular, groundwater supply, greenhouse gases reduction, and surface water quality). Alternative land cover choices indicate tradeoffs among the value of ecosystem services created and the economic returns. These tradeoffs are explored using efficiency frontiers that determine the least value in ecosystem services that must be given up to generate additional economic returns. Agricultural producers may switch to irrigation with surface water using on-farm reservoirs and tail water recovery systems in response to groundwater overdraft, and this has consequences for the bundle of ecosystem service values and economic returns achievable from the landscape. Planning that accounts for both ecosystem service value and economic returns can achieve more value for society, as does the adoption of reservoirs though lowering the costs of irrigation, increasing groundwater levels, and reducing fuel combustion and associated GHG emissions from groundwater pumping. Sensitivity analyses of per unit value of ecosystem services, crop prices, and the groundwater and water purification model parameters indicate tradeoff among ecosystems service values, such as the use of a high-end social cost of carbon ultimately lowers groundwater supply and water purification value by more than 15%.

## Introduction

In response to groundwater overdraft, increases in groundwater pumping costs, and the degradation of surface water quality from agricultural runoff, irrigation may shift from well pumping toward surface water from on-farm reservoirs with tail-water recovery systems that capture agricultural runoff. The economic and institutional aspects of conjunctive water management are well studied [1, 2], but the influence of reservoirs on groundwater supply, surface water purification, and greenhouse gas (GHG) reduction has received less attention. The choice of reservoirs can raise the aquifer volume, which in turn affects the crops grown, and the land cover decisions influence nutrient and sediment runoff and GHG emissions. We use efficiency frontiers to examine the tradeoff of the economic returns and ecosystem services for an agricultural landscape with and without reservoirs. An efficiency frontier for the landscape is made by maximizing the economic returns over the entire possible range of ecosystem service values. We investigate if on-farm reservoirs and tail-water recovery systems causes economic returns and ecosystem services to rise and how the tradeoff from the efficiency frontiers between economic and ecosystem service objectives is affected.

The conjunctive water management system we examine includes on-farm reservoirs to store abundant surface outside the irrigation season and tail-water recovery systems that brings the runoff leaving the field to the reservoir. The use of the surface water conserves groundwater and the collection of tail-water for reuse on the farm can improve surface water quality [3]. The groundwater supply, surface water purification, and greenhouse gas (GHG) reduction ecosystems services we analyze in this paper may all be affected by the use of the reservoirs and tail-water recovery. Reservoirs can reduce groundwater use and agricultural runoff, but greater rice production may release more of the potent GHG methane. Although surface water is less expensive to pump than groundwater, economic returns do not necessarily rise with reservoirs because they occupy productive land and have construction and on-going maintenance costs.

A tradeoff in ecosystem service value foregone to achieve greater economic return is observed by the slope of the efficiency frontier. The landscape that is societally optimal is where the loss of a dollar of ecosystem service value is exactly balanced by a dollar gain in economic returns. Elsewhere on the frontier, social value can be increased by moving along the frontier toward the objective that gives more value than is given up. Building on-farm reservoirs with tail-water recovery may increase the efficiency of the landscape at providing both economic and ecosystem service value, but the extent that the frontier shifts outward is the empirical focus of this study.

Irrigation in the Lower Mississippi River Basin in Arkansas (also known as Arkansas Delta) comes principally from groundwater within the Mississippi River Valley Alluvial Aquifer. The overdraft of the aquifer means that by 2050 agricultural demand for groundwater will exceed available supplies by 7 million acre-feet per year [4]. We optimize farm net returns over time to create the efficiency frontier for a farm landscape with spatially specific sites by changing the extensive crop margin (e.g. a shift away from irrigation intensive rice toward non-irrigated soybeans) and the irrigation water source (e.g. reservoir or well). When we optimize the farm net returns, we hold the total value of the ecosystem services fixed at a particular level, with ecosystem services put in monetary terms using their estimated social value, to create a point on the efficiency frontier. A low total value of ecosystem services required for the landscape means that crops with high economic value that are heavily polluting or irrigation intensive can be grown. However, as the total value of ecosystem services required for the landscape increases, then land covers that are less polluting and irrigation intensive will have to occupy the landscape instead of the high value crops, which lowers the economic returns.

The challenge is to create ecosystem service models that accurately represent the value of the ecosystem services associated with the crop and irrigation decisions in sufficient scientific detail while also being able to solve an optimization model to create the efficiency frontiers. We model changes in groundwater supply by tracking the aquifer’s saturated thickness in response to well pumping, and this depends principally on the hydro-conductivity of the groundwater flow and the proximity to nearby wells whose pumping alters the groundwater flow. The precipitation, evapotranspiration, the slope of the land, and the tillage and irrigation practices affect soil and nutrient runoff that can reduce the quality of surface waterbodies. The relationship between the land cover and pollutant loading of the surface water depends on a digital elevation model that routes water downhill to the streams. Farm tillage practices, soil type, and fuel combustion from irrigation pumping all influence the release of GHGs from land cover of the agricultural landscape.

Much of the prior work that looks at the tradeoffs among multiple environmental objectives and economic returns does not use efficiency frontiers [5, 6, 7, 8, 9]. These studies examine bundles of land use that generate different services such as an intensive agricultural landscape producing high levels of agricultural products but low water quality and carbon storage. Without efficiency frontiers, the bundles of land cover are illustrative of the tradeoff among ecosystem services, but they do not reveal the required tradeoffs to obtain more of one service at the least loss to the other. Studies that look at how groundwater depletion affects water quality and economic returns from an agricultural landscape do not consider the tradeoff with GHG reductions [10].

However, a growing strain of literature is using efficiency frontiers to analyze the optimal tradeoffs among different ecosystem services or between ecosystem services and economic returns. Nelson et al. [11] use efficiency frontiers to examine the tradeoff between carbon sequestration and species conservation use, but not economic returns. The efficiency frontier tradeoff between species conservation, but not ecosystem services, and economic returns is considered in Polasky et al. [12]. This literature on the efficiency frontiers does not focus on the agricultural landscape or the tradeoffs among economic returns and groundwater supply, surface water quality, and GHG reduction value. Nor has any prior work focused on how private irrigation infrastructure investment such as conjunctive water management influences the efficiency frontier.

## Methods

This section describes the model components for creating the efficiency frontiers that examine the tradeoffs among economic returns and ecosystem services. The land cover is the basis for computing the economic returns and ecosystem services from the agricultural landscape. The economic model uses the land cover and irrigation decisions of the agricultural producer to maximize farm net returns. The ecosystem service model uses the land cover and irrigation decisions to calculate the water supply value, water purification value, and the GHG reduction value. We conclude the methods section with a description of how the economic and ecosystem service models are used to create the efficiency frontiers, the sensitivity analyses applied to the model parameters for calibration, and the conservation policies evaluated.

The land cover of the farm landscape includes crops, reservoirs, and conserved land set aside through a rental program of the government. The chosen crops generate economic returns, but irrigation depletes groundwater. Also, agricultural runoff pollutes surface water, and farm production activities release GHGs. The landscape is spatially heterogeneous due to differences in long term investment in farm practices, soil types, and access to water resources. A thirty-year time horizon *T* is chosen for a single generation of farmers to observe how depletion of the aquifer influences production decision, and a grid of *m* cells (sites) represents spatial differences.

### Land cover

The major crops include flood irrigated rice, and furrow irrigated soybean, corn, and cotton, non-irrigated sorghum and soybean, and double cropped irrigated soybean with winter wheat. Arkansas has average annual precipitation amounts ranging from approximately 50 to 57 inches per year, and has often been considered an area rich in water resources [13]. However, there is a lack of timely rainfall and an increasing need for irrigation, which has spurred the growing use of reservoirs with tail-water recovery systems [14]. There are *n* possible land cover types *j* at the end of period *t* as denoted by *L*_*ijt*_ for site *i* that include each of the crops, reservoirs that have tail-water recovery, and the US Department of Agriculture’s Conservation Reserve Program (CRP). At the end of each annual period *t*, we assume any land cover *j* can become another land cover or on-farm reservoirs with tail-water recovery, except for CRP which cannot transition to another cover until a ten-year contract period has elapsed. A profit maximizing farmers may switch land out of irrigated crops into non-irrigated crops with declining groundwater availability at the end of each period.

The initial land availability equals the sum of the land covers chosen for site *i* at any time *t* (Eq 1).

$$\sum_{j}^{n}L_{ijt}=\sum_{j}^{n}L_{ij0},\text{ for }j=\text{ crops},\text{ CRP},\text{ on-farm reservoirs}$$ (1)

#### The economic model

The net present value of the agricultural production over all time periods and the entire landscape is the economic objective. We describe the irrigation components of the economic model first followed by the economic objective that the agricultural producers optimize.

#### Irrigation

The average annual irrigation that crop *j* receives to supplement precipitation, *wd*_*j*_, is the demand for irrigation in acre-feet. The groundwater stored in the aquifer beneath site *i* at the end of the period *t* is *AQ*_*it*_. The water that comes from the on-farm reservoirs is *RW*_*it*_, and the water from well pumping is *GW*_*it*._ There is recharge of the groundwater, *nr*_*i*_, that occurs naturally from precipitation, streams, and underlying aquifers each period.

Eq (2) shows the acre-feet of water stored in an acre reservoir [10] as

$$\left(\omega_{\text{max}}+\omega_{\text{min}}\right)-\frac{\omega_{\text{max}}}{\sum_{j}^{n}L_{ij0}}L_{iRt},$$ (2)

which includes, *L*_*iRt*_, as the acres in reservoirs at time *t*, and the total acreage at site *i*, $\sum_{j}^{n}L_{ij0}$. The size of the reservoir influences the volume of water that each acre of the reservoir holds in a year because there is a fixed amount reservoir water at each site that comes from precipitation and from agricultural runoff due to irrigation collected through tail-water recovery. If the reservoir occupies the entire site *i* and only the rainfall fills the reservoir, then the low-end acre-feet of water that fills each reservoir acre is *ω*_min_. If the reservoir is less than the size of the site, then recovery of the runoff and rainfall fills the reservoir to a high-end capacity in acre-feet per reservoir acre of (*ω*_max_ + *ω*_min_). Reservoirs are likely placed on land that is relatively less productive for agriculture. Through the model optimization, the least profitable land would be replaced by reservoirs, but the effect of soil differences within sites on the crops grown is not modeled. Variations in crop yield across sites mean that the sites with worse soil and lower yields will have more reservoirs. We do not account for within growing season variability in evaporation, leakage, and the timing of rainfall, which could influence *ω*_max_ and *ω*_min_ for the reservoir.

The intensity of well pumping across the landscape influences aquifer depletion over space. The proportion of the underground flow into the aquifer at site *k* and out of site *i* when an acre-foot is pumped from a well at site *k* is *p*_*ik*_, which depends on the distance and the lateral speed of underground water movement based on the soil profiles observed between sites. Jenkins [15] quantifies the groundwater flow out of site *i* in response to a nearby pump at site *k* as the hydraulic diffusivity of the aquifer divided by the square of the shortest distance between the pumped well and the nearby site. The groundwater flow out of site *i* into site *k* divided by the sum of all groundwater flow out of site *i* is the how the proportion *p*_*ik*_ is calculated. This means the groundwater that leaves site *i* is $\sum_{k}^{m}p_{ik}GW_{kt}$. We assume pumps operate with the same efficiency and power units to deliver a fixed amount of water per minute.

The water used for irrigation must be less than the water available from reservoirs and wells Eqs 3 and 4 indicates the water stored in the reservoirs must be greater the water used from the reservoirs. The aquifer volume in the previous period less the spatially weighted proportion of water pumped from the surrounding sites plus natural recharge equals the current aquifer volume (Eq 5). The cost of pumping groundwater at a site, *GC*_*it*_, depends on the cost to lift an acre-foot of water by one foot, *c*^*p*^, and the initial depth to the groundwater, *dp*_*i*_. The depletion of the aquifer volume, (*AQ*_*i*0_ − *AQ*_*it*_), divided by the area of the site, $\sum_{j}^{n}L_{ij0}$, shows how much the depth to the aquifer increases. Capital costs per acre-foot for the well, which accounts for new well drilling in response to aquifer decline, is *c*^*c*^ (Eq 6).

$$\sum_{j=1}^{n}wd_{j}L_{ijt}\leq GW_{it}+RW_{it}$$ (3)

$$RW_{it}\leq \left(\left(\omega_{\text{max}}+\omega_{\text{min}}\right)-\frac{\omega_{\text{max}}}{\sum_{j}^{n}L_{ij0}}L_{iRt}\right)L_{iRt}$$ (4)

$$AQ_{it}=AQ_{i(t-1)}-\sum_{k}^{m}p_{ik}GW_{kt}+nr_{i}$$ (5)

$$GC_{it}=c^{c}+c^{p}\left(dp_{i}+\frac{\left(AQ_{i0}-AQ_{it}\right)}{\sum_{j}^{n}L_{ij0}}\right)$$ (6)

#### Economic objective

The cost to produce an acre of the crop excluding the irrigations costs *ca*_*j*_ and the price per conventional unit of the crop is *pr*_*j*_ are constant in real terms. We assume no productivity growth trend for the constant yield of crop *j* per acre at site *i*, *y*_*ij*_. Excluding the costs of irrigation, the net value for crop *j* is then *pr*_*j*_*y*_*ij*_—*ca*_*j*_ per acre. The CRP payment per acre to the landowner, *pr*_*crp*_*y*_*icrp*_, with yield normalized to one and price is the payment per acre, less the cost to establish and maintain an acre of CRP (*ca*_*crp*_) is net value per acre of CRP. The reservoir pumping cost per acre-foot is *c*^*rw*^, and the per acre capital and maintenance cost of a reservoir each period is *c*^*r*^. We make values over time comparable in monetary terms using the real discount factor, *δ*_*t*_.

Eq 7 indicates the economic objective to maximize the present value of farm profits over the fixed horizon *T* by changing the amount of land in each crop or CRP, the reservoir water use, and groundwater use, namely *L*_*ijt*_, *RW*_*it*_, and *GW*_*it*_. The choice of the thirty-year fixed time horizon is chosen to represent the planning for a single generation of agricultural producers, and the time horizon does influence the results such that a shorter horizon leads to faster annual depletion of the aquifer and a longer horizon leads to slower annual depletion of the aquifer. The initial condition of the state variables and the non-negativity constraints on land, water use, and the aquifer are shown in Eqs 8 and 9.

$$\underset{L_{ijt},RW_{it},GW_{it}}{\text{max}}:\sum_{t=1}^{T}\delta_{t}\left(\sum_{i=1}^{m}\sum_{j=1}^{n}\left(pr_{j}y_{ij}-ca_{j}\right)L_{ijt}-c^{r}L_{iRt}-c^{rw}RW_{it}-GC_{it}GW_{it}\right)$$ (7)

Subject to:

$$L_{ij0}=L_{0}^{ij},L_{iR0}=0,AQ_{i0}=AQ_{0}^{i},$$ (8)

$$L_{ijt}\geq 0,RW_{it}\geq 0,GW_{it}\geq 0,AQ_{it}\geq 0.$$ (9)

and the spatial dynamics of land and irrigation (Eqs 1–6). The crop and irrigation choices from the optimization of Eq 7 influence ecosystem services related to GHGs, water purification, and groundwater availability, but they are not directly considered by producers.

#### The ecosystem service model

We track changes in the physical ecosystem services over time, and the social value of these ecosystem services determine the monetary value of the changes in ecosystem services to calculate the net present value of ecosystem services from the landscape. The description of the model for calculating greenhouse gases reduction value is first followed by a description of the model components for the water purification value and the groundwater supply value.

#### Greenhouse gases reduction value

GHG emissions per acre of vegetation on a land cover are associated with the production of crops and CRP for the major production practices of the Arkansas Delta based on a life cycle assessment (LCA) up to the farm gate [16]. Fuel use and emissions generated during the manufacture of chemicals and fertilizer, methane emissions from rice production, and nitrous oxide emissions from the application of nitrogen fertilizer to soil are tracked in carbon equivalents (CE) in kg per acre for land cover *j* (*E*_*j*_). Pumping ground and reservoir water releases fuel combustion emissions, and the range of irrigation emissions is in Figure A.1 in S1 Supporting Information. The depth of the well multiplied by a conversion factor *σ*_*g*_ that identifies the carbon emitted from fuel combustion to lift an acre-foot of water one foot and multiplied by the acre-feet of groundwater pumped indicate the emissions from groundwater pumping at site *i*, *EG*_*it*_. The acre-feet of reservoir water pumped multiplied by a conversion factor *σ*_*r*_ for the carbon emitted from fuel combustion to pump an acre-foot of water into a reservoir and back out to the field is the emissions from pumping reservoir water at site *i*, *ER*_*it*_. The total carbon emissions for time *t* at site *i* (*E*_*it*_) is shown in Eq 10 as

$$E_{it}=\sum_{j}^{n}E_{j}L_{ijt}+EG_{it}+ER_{it}.$$ (10)

Aboveground biomass (*AGB*_*ij*_) and belowground biomass (*BGB*_*ij*_) sequester carbon, with the details of this in S1 Supporting Information, and this sequestration depends on the soil texture and tillage practices [17]. A weighting of soil textures at each site *i* determines the soil factor, *ξ*_*i*_, which is the fraction of carbon lost to respiration due to soil related microbial activity. Porous soil (i.e. sandy) has more intense wetting and drying cycles, and this encourages microbial activity and respiration compared to finer textured soils (i.e. clay). Eq 11 tracks the carbon sequestration, *S*_*it*_, for time *t* at site *i* as

$$S_{it}=\sum_{j}^{n}\left[\left(AGB_{ij}+BGB_{ij}\right)\xi_{i}\right]L_{ijt}.$$ (11)

Although the sequestration is likely to be greater initially and slower later on CRP land [18], we suppose sequestration occurs evenly over time. Eqs 10 and 11 constrain the ecosystem services objective but do not influence the economic returns objective.

The cost to society incurred by the predicted damages from each additional ton of carbon equivalent emitted to the atmosphere is the social cost of carbon, *p*_*c*_, and this indicates the monetary value of a ton less of carbon equivalent GHGs from the agricultural landscape [19]. Eq 12 says the value of avoided damages, *V*_*c*_, is negative if the emissions outweigh sequestration (Eq 12).

$$V_{c}=\sum_{t=1}^{T}\delta_{t}\left(\sum_{i}^{m}p_{c}\left(S_{it}-E_{it}\right)\right).$$ (12)

#### Water purification value

We use the Integrated Valuation of Ecosystem Services and Tradeoffs (InVEST) [20] water purification model to estimate sediment, phosphorus, and nitrogen runoff from the initial land cover. Natural land, urban areas, public land, and lakes are in the water purification model, although not part of the land cover in the optimization model, because they affect the agricultural runoff from each site that reaches streams. Based on soil characteristics, precipitation, slope, and evapotranspiration, the expected annual water yield at each site is calculated. The expected pollutant loading and the filtering capacities for the initial land cover is combined with the water yield to calculate the pollutants from each site that eventually reach a stream. Using the nutrient and sediment pollution from the initial land cover, we calibrate these exports from the initial land cover, using the pollutant loading and filtering capacities, to generate the pollutant *k* export per acre from land cover *j* for farm site *i* that reaches a stream, *P*_*ijk*0_.

The values for *P*_*ijk*0_ are used to calculate how the pollutant loading to the mouth of the watershed change in response to the land cover transitions. We assume pollutant exports from site *i* are associated only with the land cover changes at site *i* but not with land cover transitions at surrounding sites. Assuming no spatial interactions of land cover transitions with surrounding sites is necessary to have the optimization problem to solve. These spatial interactions may have potentially large second order effects that are worth further investigation but are outside the scope of the paper here. Tail-water recovery systems have the ability to capture runoff and prevent this from reaching natural streams, but the slope at site *i* affects the effectiveness of the system. The tail-water recovery system effectiveness, 0 ≤ *θ*_*i*_ ≤ 1, is greater if site *i* is flatter [21].

Eq 13 indicates the amount of pollutant *k* reaching the mouth of a watershed from each site *i* at time *t* (*EX*_*ikt*_) as

$$EX_{ikt}=\sum_{j}^{n}P_{ijk0}L_{ijt}\left(1-\theta_{i}\frac{L_{iRt}}{\left(L_{iRt}+1\right)}\right),$$ (13)

where *P*_*ijk*0_*L*_*ijt*_ is the export without reservoirs of the pollutant *k* to a stream from site *i* and land cover *j*. A site with reservoirs has a value less than one for $\theta_{i}\frac{L_{iR\_t}}{\left(L_{iR\_t}+1\right)}$ because there is land in *L*_*iR_t*_, and this reduces the export of pollutants to streams.

The willingness to pay (WTP) per household for a water purification improvement (*wtpq*_*b*_) depends on the baseline water quality and median household income of the basin *b*. The WTP values per household are prorated to the percent reduction in the pollutant *k* loading from all sites *i* in basin *b*, $\left(\frac{\sum_{i\in b}\left(EX_{ikt}-EX_{ik(t+1)}\right)}{\sum_{i\in b}EX_{ikt}}\right)$. In this instance, we use $\sum_{i\in b}\left(EX_{ikt}-EX_{ik(t+1)}\right)$ because a fall in pollutant exports corresponds to an increase in water purification value. Multiplying the number of households in the basin (*hh*_*b*_) by the prorated WTP per household for pollutant *k* is the present value of the surface water purification, *V*_*w*_, shown as Eq 14

$$V_{w}=\sum_{t=1}^{T}\delta_{t}\left(\sum_{k}\sum_{b}hh_{b}wtpq_{b}\left(\frac{\sum_{i\in b}\left(EX_{ikt}-EX_{ik(t+1)}\right)}{\sum_{i\in b}EX_{ikt}}\right)\right).$$ (14)

#### Groundwater supply value

We consider only the groundwater value to agricultural producers to buffer against periodic shortages in surface water supplies, *p*_*bv*_, because there is inadequate data to estimate the damages from subsidence and losses to in-stream flows. Aquifer volume falls if the natural recharge of the aquifer is less than the groundwater withdrawal for irrigation. Eq 15 indicates the present value of the groundwater buffer value, *V*_*g*_, as

$$V_{g}=\sum_{t=1}^{T}\delta_{t}\left(\sum_{i}^{m}p_{bv}\left(AQ_{i(t+1)}-AQ_{it}\right)\right).$$ (15)

#### Ecosystem services objective

The sum of the present value of GHG reduction, surface water purification, and groundwater buffer value is the ecosystem services objective (Eq 16). The objective is to maximize the present value of ecosystem services by determining *L*_*ijt*_, *RW*_*it*_, and *GW*_*it*_ over the fixed time horizon *T*

$$\underset{L_{ijt},RW_{it},GW_{it}}{\text{max}}:\;V_{c}+V_{w}+V_{g},$$ (16)

subject to the Eqs 1–6, 10, 11 and 13. The crop and irrigation choices from the optimization of Eq 16 influence farm profits but they are not directly considered by conservation planners.

#### Efficiency frontier

We trace out an efficiency frontier, showing the tradeoff of ecosystem service value and economic returns, by finding the maximum economic returns for a fixed value of an ecosystem service, and then varying the fixed value of the ecosystem service over its entire potential range. We compare how the reservoirs affect the shape and position of the efficiency frontier by finding efficiency frontiers without and with reservoirs. The efficiency frontier illustrates the greatest economic return and ecosystem service values feasible on the landscape and the necessary reduction in ecosystem service value to increase economic returns from the landscape.

By optimizing the ecosystem services objective without a restriction on economic returns, the maximum value of the ecosystem services is found. Conversely, by optimizing economic returns without restriction on the ecosystem service value, the minimum value of the ecosystem services is found. Next, ecosystem service values are chosen that extend for minimum and the maximum ecosystem service values range to trace out the shape of the frontier. Lastly, for each levels of ecosystem service value from the previous step, we maximize economic returns. A combination that rests on the efficiency frontier is an economic returns maximum that corresponds to a given ecosystem service value.

Ecosystem service values for the efficiency frontier with reservoirs are chosen because they match the ecosystem service values chosen for the efficiency frontier without reservoirs. A determination of the gains from moving to an outer frontier is possible by using the same ecosystem service values across frontiers. We trace out the rest of the frontier with reservoirs by choosing evenly spaced ecosystem service values. We use the non-linear programming solver CONOPT from AKRI Consulting and Development to perform the optimization in the Generalized Algebraic Modeling System (GAMS).

#### Sensitivity analyses and conservation policies

Three sensitivity analyses examine the responsiveness of the economic returns optimization to the economic and ecosystem parameters. These include looking at low- and high-end i) social values for the GHGs, groundwater supply, and water purification, ii) prices for rice, soybeans, and corn, and iii) the hydro-conductivity of the aquifer and the nutrient loadings from all the crops. The sensitivity analyses involve maximizing economic returns to see how crops and ecosystem services respond to the parameter changes while maintaining total ecosystem service value at a particular level on the efficiency frontier without reservoirs.

Conservation policies are meant to align profit making decisions with the provision of ecosystem services. We compare the model of the economic returns objective with reservoirs and no conservation policy to the conservation policies that include 65% cost-share on reservoir construction costs based on the rate from Natural Resource Conservation Service’s Agricultural Water Enhancement Program [22], tax on groundwater pumping costs of 15% is chosen to achieve groundwater conservation similar to the cost share on reservoir construction, a total maximum daily load of phosphorous and sediment chosen as the phosphorus and sediment exports from point L on the efficiency frontier without reservoirs, and a policy that has the value for carbon credits at $28.51 per metric ton of carbon according to the clearing price of the March 2015 auction by the European Union Emission Trading Scheme and an exchange rate of $0.87 per euro [23].

The policies for the cost-share on reservoir costs and groundwater taxes result in transfers between the government and producers while the carbon credits policy cause transfers among producers. All the policies make the economic returns less transfers from the government fall, and this is an economic cost to society because the policy directs production decisions away from maximum economic returns. The economic cost per dollar of ecosystem service value gained is calculated as the difference in economic returns without and with the policy and dividing this by the difference in total ecosystem service value with and without the policy.

## Data

**Fig 1:**
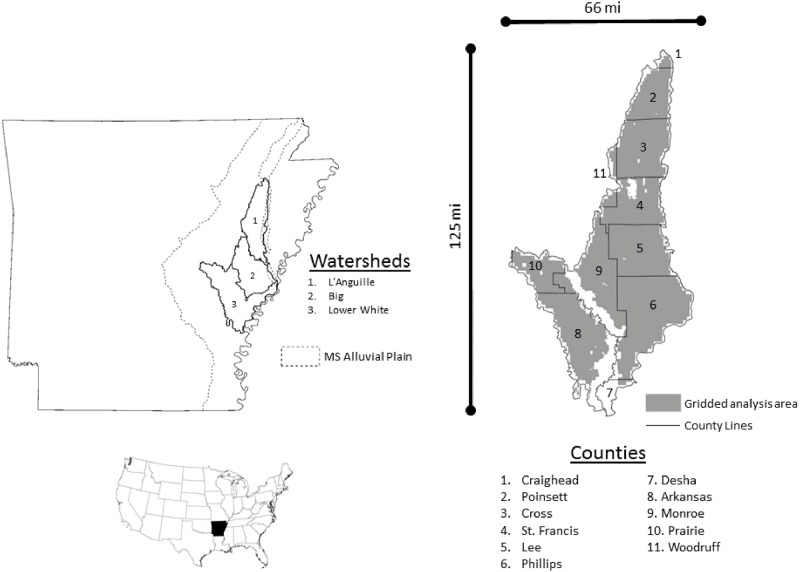
Mississippi Delta Watersheds. Three eight-digit hydrologic unit code (HUC) watersheds in the Mississippi Delta region of eastern Arkansas define the outer boundary of the study area. An eight-digit HUC defines the drainage area of the sub-basin of a river. County lines overlay the study area. Public land and urban areas are excluded. The location of the study area within the State of Arkansas is shown.

**Fig 2:**
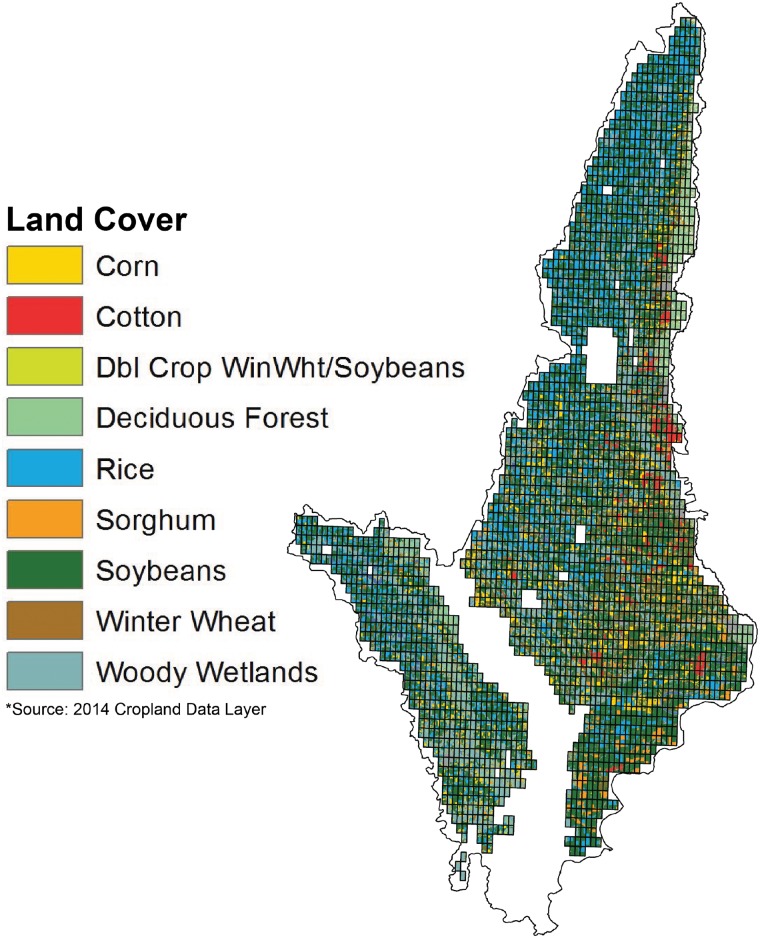
Land Coverage Grid. The grid that overlays the study area for use in modelling spatial heterogeneity is enlarged. The crops and other land covers initially present in each grid cell are shown from the 2014 Cropland Data Layer.

The outer boudary of the study area consists of three eight-digit hydrologic unit code watersheds in the Arkansas Delta region with critical groundwater areas and non-point source pollution priorities (Fig 1). These watersheds overlap eleven Arkansas counties, and the average for the past 5 years of crop yields by county is a proxy for the yield of the crops [24]. We evaluate crop mix and irrigation methods on a landscape with spatial heterogeneity by dividing the study area into a grid of 2,724 sites (Fig 2). The sites are six hundred acres in size to represent the average farm size for the region. Sites having entirely non-cropland uses, e.g. public lands water, and urban areas, in the 2014 Cropland Data Layer (CDL) are removed [25]. The initial acreage of rice, corn, cotton, soybeans, and sorghum comes from the 2014 CDL, and on the basis of harvested acreage for 2010–2011 the soybean acreage with each site is split into non-irrigated soybean, irrigated soybean, and double crop soybeans based on county level statistics (Table A.1 in S1 Supporting Information) [26]. The yield of the 30yr Treasury Bond over the last decade [27] suggests a real discount rate of 5%.

### Aquifer

Table A.1 in S1 Supporting Information shows the initial depth to the water table and saturated thickness of the Alluvial aquifer from the Arkansas Natural Resources Commission [4]. The acreage of the site times the saturated thickness of the aquifer is the volume of the aquifer at site *i*. Precipitation and the flow from streams and the underlying Sparta aquifer influences the natural recharge (*nr*_*i*_) [28].

Well pumping reduces the aquifer below the pumped well and for the sites that surround the well. In response to well pumping, some of the aquifer flows from the surrounding sites into the site with the pumped well. The lateral flow of water that determines the spatial weight (*p*_*ik*_) is based on the hydraulic diffusivity of the aquifer divided by the square of the shortest distance between the pumped well and the nearby site [15]. The ratio of the transmissivity and the specific yield of the unconfined aquifer is the hydraulic diffusivity [29]. The dimensionless ratio of water drainable by saturated aquifer material to the total volume of that material is the specific yield. Transmissivity is the product of hydraulic conductivity and saturated thickness, and hydraulic conductivity is the rate of groundwater flow per unit area under a hydraulic gradient [29].

Spatially coarse pilot points digitized in Clark, Westerman and Fugitt [30] provide the hydraulic conductivity in feet per day for the Mississippi River Valley alluvial aquifer. The average hydraulic conductivity is 226 feet per day for the study area sites. For the sensitivity analysis on the low- and high-end hydraulic conductivity, the hydraulic conductivity of all sites is replaced with the highest and lowest hydraulic conductivity in the study area, which is 39 and 413 feet per day, respectively. The data and model for the aquifer are described in more detail above Table A.1 in S1 Supporting Information.

A stable supply of groundwater represents an irrigation stabilization value called buffer value [31]. Based on the variability of seasonal rainfall, the curvature of the soybean yield response to water, and the net profit of soybeans, we estimate the buffer value of an acre-foot of groundwater as $5.19 [32]. The stabilization or buffer value of groundwater depends on the variability of rainfall, which could be affected by climate change, and the steepness of the derived demand for water at the average surface rainfall, which depends on the price of the crop and the water requirements of the crops. Using the variability in the net profit of soybeans based on the future prices over the last five years [33], we estimate the low- and high-end buffer values for the groundwater as $0.72 and $13.82 per acre-foot.

### Farm production and the on-farm reservoir and tail-water recovery system

The costs of production by crop from the 2014 Crop Cost of Production estimates, excluding irrigation, are shown in Table A.2 in S1 Supporting Information [24]. More detail on the variable irrigation costs are described before Table A.2 in S1 Supporting Information. The crop specific irrigation water use comes from the Division of Agriculture [24]. The five-year average of December futures prices for harvest time contracts for all crops are used for the crop prices [13]. The low- and high-end prices for rice, soybean, and corn are the lowest and highest December future prices for those crops over the last five years. The sign-ups in Arkansas as of March 2015 indicate the CRP payment per acre [34].

The minimum volume of water (ω_*min*_) an acre reservoir will hold comes from the tail-water recovery system collecting rainfall alone to fill a reservoir by 1.4 acre-feet of water [35]. The maximum capacity accounting for evaporation of 11 acre-feet per acre is based on irrigation runoff supplementing the rainfall runoff [36]. The average share of nutrients and sediment captured by reservoirs (*θ*_*i*_) varies according to the slope of each site *i* [37], and this is about 0.87 [3]. More detail about on-farm reservoir/tail-water recovery construction and maintenance costs are described before Table A.2 in S1 Supporting Information.

### Water purification and greenhouse gases

A digital elevation model directs surface water downhill in a geographic information system (GIS). As the water travels over each site, the site either subtracts or adds depending on the land cover, to the quantity of the nutrient (Table A.3 in S1 Supporting Information) and sediment (Table A.4 in S1 Supporting Information) that reaches a stream. The quantity of nutrients leaving each site depend on the export and filtering characteristics of each land cover, the water yield (which is the difference of precipitation and evapotranspiration), and the slope of the land. The sediment transport to a stream follows a universal soil loss equation, which uses rainfall erosivity, soil erodibility, slope gradient factor, crop management factor such as tillage, and support practices such as cross slope versus downslope furrows. The cumulative loading of the nutrients and sediment to the mouth of a watershed is the export from all the sites that reach streams in that watershed. Low- and high-end nutrient loading scenarios are based on 25% lower and higher export coefficients for nitrogen and phosphorous for all the land covers based on the standard deviations of these export coefficients found in the literature (Table A.3 in S1 Supporting Information). More details on the water purification model are in S1 Supporting Information. An average willingness to pay (WTP) value per household per year of $49.94 for a 20% reduction in pollutant loadings is reported by Hite et al. [38]. Based on the reported standard deviation in the WTP for a 20% reduction in nutrient loadings, we use a low- and high-end WTP of $39.51 and $60.38 per household per year. The multiplication of the household WTP prorated to the percentage change in loadings at the mouth of each watershed and the projected number of households in the basin [39] gives the WTP per basin in each period.

Using the production estimates from crop enterprise budgets [24], we track greenhouse gas emissions from fuel, fertilizer, and chemical applications. We track above and below ground biomass production [17] with county level yields to determine soil carbon sequestration. Plant residue left in the soils becomes a fraction of carbon after microbial decomposition and gas fluxes (Table A.5 in S1 Supporting Information). Further adjustment to carbon sequestration occurs based on tillage and soil texture. Emissions from irrigation fuel combustion change in response to the model outcome for the depth to the aquifer. The reduction of GHGs in the atmosphere creates value for society because of the lower expected damages from climate change. Based on the fitted median distribution and a 1% pure rate of time preference from Tol [19] and after adjusting to 2015 dollars, the constant real estimate is $134 per ton carbon ($35.57 per ton CO_2_). The low- and high-end social value of carbon abatement is $66 and $209 per ton carbon based on the 33^rd^ and 67^th^ percentiles for the fitted distribution and 1% pure rate of time preference.

## Results

**Fig 3:**
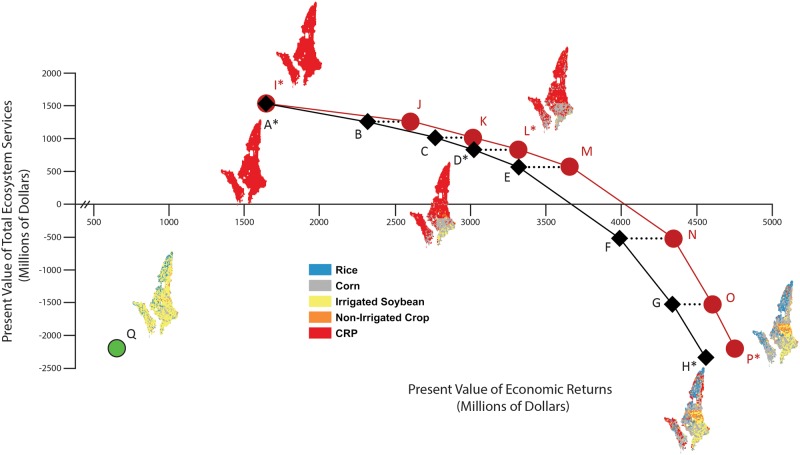
Value of Ecosystem Services Versus Economic Returns. Crop mix patterns associated with specific points along the efficiency frontiers without reservoirs (Points A to H) and with reservoirs (Points I to P). Point Q indicates what the continuation of current landscape throughout the study period would mean for the present value of total ecosystem services and economic returns. Each crop mix pattern shown beside the efficiency frontiers corresponds to a lettered point with an asterisk on the frontiers.

Two efficiency frontiers (Fig 3) examine the tradeoff of ecosystem service value necessary to increase economic returns. The efficiency frontiers include one without on-farm reservoirs (Points A to H) and one with on-farm reservoirs (Points I to P). Going from Point A along the curve toward Point H, the required ecosystem service value falls, and this allows crops with greater economic return to be grown. The change in crops to raise economic returns cannot result in a lower ecosystem service value than is required for that position on the frontier. This means that large crop increases into high value rice cannot occur halfway along the frontier because the fall in GHG value would cause the total ecosystem service value to fall below the required value.

Point A in Fig 3, where all the land is put into CRP, generates the maximum value for all the ecosystem services. The rental payments by the government for CRP mean the landscape at Point A also has positive economic returns. Moving from point A to point D increases economic returns by 83% while reducing the value of all ecosystem services by 47% (see Table 1). The switch from CRP to irrigated corn and non-irrigated crops raises economic returns (see Table 2). Greenhouse gas value declines by 46%, and the groundwater supply value and water quality value decline by 55% and 34%, respectively. Going around the efficiency frontier from point D to point H shifts nearly all land into irrigated production, and this increases economic returns by 51% while ecosystem services decline by 386%. Rice acreage increases the most with the move to point H as well as significant increases in corn and irrigated soybean. The combined value of economic returns and all ecosystem services is higher at Point D than at Point H.

**Table 1:** Ecosystem service and economic return values for points along efficiency frontiers.

Without reservoirs			With reservoirs		
Efficiency frontier points	Present value of economic returns	Present value of ecosystem services	Efficiency frontier points	Present value of economic returns	Present value of ecosystem services
A	1649	1532	I	1649	1532
B	2331	1253	J	2616	1253
C	2768	1000	K	3021	1000
D	3021	819	L	3322	819
E	3322	567	M	3656	567
F	3995	-511	N	4356	-511
G	4322	-1522	O	4693	-1522
H	4559	-2345	P	4757	-2206

Note: The values of economic returns and ecosystem service values are reported in millions of 2015 constant dollars.

**Table 2:** Present value of economic returns and ecosystem services for select points on the efficiency frontier using all ecosystem service value (in millions of 2015 constant dollars).

Ecosystem service or land cover	Without reservoirs			With reservoirs		
	A	D	H	I	L	P
Greenhouse gases	1439	771	-2276	1439	760	-2147
Groundwater supply	60	27	-68	60	41	-56
Water purification	32	21	-1	32	19	-2
Total ecosystem services	1532	819	-2345	1532	819	-2206
Rice	0	0	769	0	0	932
Irrigated soybeans	0	121	674	0	21	698
Non-irrigated crop	0	543	625	0	477	587
Corn	0	1300	1940	0	1953	2081
Cotton	0	54	430	0	21	439
CRP	1649	1003	121	1649	851	20
Total economic return	1649	3021	4559	1649	3322	4757
Total of economic return and ecosystem service value (Value to society)	3181	3840	2214	3181	4141	2551

The availability of reservoirs does not affect the maximum ecosystem services because a landscape entirely in CRP provides the greatest ecosystem service value. By moving from point I to point L, economic returns increase by 101% because irrigated crops be grown at a lower cost because of less well pumping (see Table 2). Comparing the maps of points D and L in Fig 3, reservoirs increase corn on the landscape in the southern and eastern sites of the study area. Movement from point L to point P shifts more CRP land into rice and irrigated soybeans in the western and northern sites where groundwater is relatively scarce. The move to point P raises the economic returns by 43%, but ecosystem services fall by 369%. The value of ecosystem services at point P is higher than at point H because reservoirs conserve more groundwater and more GHG sequestering corn is grown. The landscape at Point L achieves a higher value for society than a landscape managed for economic returns alone (Point P).

The prices farmers receive for a crop influence which crops are grown to maximize economic returns when the total ecosystem service value cannot fall below the value at Point D (Table 3). High-end prices to rice change the crops grown so that rice is grown on the landscape and more CRP and non-irrigated crops, but less corn, soybeans, and cotton are grown. The consequence of this is that groundwater supply value and greenhouse gas value falls while the water purification value rises. Economic returns rise by about 1%. The low-end value for rice price has no influence on the crops grown or the associated ecosystem service values because Point D also has no rice on the landscape. With the high-end price for soybeans, more soybeans are grown instead of the non-irrigated crop while the other crops and CRP are largely unchanged. The groundwater supply value falls slightly from the expansion of soybeans, but there is a rise in GHG value. The opposite occurs for the low-end price to soybeans, where less soybeans and non-irrigated crop mean slightly higher groundwater supply value and slightly lower GHG value. The economic returns change by less than 1% in response to the low- and high-end price to soybeans. Corn prices at the high-end result in more corn, much less non-irrigated crop, and slight declines in soybeans, cotton, and CRP. The associated ecosystem service changes are less groundwater supply value and water purification value but more GHG value. With the low-end corn price, there is a rise in the non-irrigated crop and a fall in corn while CRP and other crops increase slightly. Since corn occupies a greater part of the landscape, the economic returns rise and fall by more than 1% in response to the high- and low-end prices to corn.

**Table 3:** Sensitivity of the present value of economic returns and ecosystem services without reservoirs to the price for rice, soybean, or corn relative to point D on the efficiency frontier without reservoirs (in millions of 2015 constant dollars).

Ecosystem service or land cover	Point D on the efficiency frontier without reservoirs	Rice price		Soybean price		Corn price	
		Low end	High end	Low end	High end	Low end	High end
Greenhouse gases	771	771	770	770	772	769	775
Groundwater supply	27	27	26	28	26	28	25
Water purification	21	21	23	21	21	22	19
Total ecosystem services	819	819	819	819	819	819	819
Rice	0	0	15	0	0	0	0
Irrigated soybeans	121	121	120	83	163	123	119
Non-irrigated crop	543	543	552	552	515	561	505
Corn	1300	1300	1299	1302	1299	1255	1396
Cotton	54	54	50	55	52	54	49
CRP	1003	1003	1008	1005	1001	1009	997
Total economic return	3021	3021	3044	3011	3030	3002	3066
Total of economic return and ecosystem service value (Value to society)	3840	3840	3865	3830	3849	3002	3885

The crops chosen to maximize the economic returns for the required total value of ecosystem services at Point D depend on the per unit social value of each ecosystem service (Table 4). A high-end value for the social cost of carbon increases the land in all crops except for cotton. It is easier to meet the required total ecosystem service value at Point D when the social cost of carbon is larger, and this allows higher value crops to be grown. The economic returns for the landscape with the high-end value for the carbon rises by 6% compared to Point D. The increase in irrigated corn and soybeans diminish the groundwater supply and the water purification value while the greenhouse gas value rises so that the total value of ecosystem services is the same as at Point D. The landscape with the low-end value of carbon has more cotton and less CRP and other crops, causing economic returns to fall by 4%, and the groundwater supply and water purification value rises as the greenhouse gas value falls.

**Table 4:** Sensitivity of the present value of economic returns and ecosystem services to the per unit value of the ecosystem services relative to point D on the efficiency frontier without reservoirs (in millions of 2015 constant dollars).

Ecosystem service or land cover	Point D on the efficiency frontier without reservoirs	Social cost of carbon ($/tC)		Groundwater buffer value ($/acre-foot)		WTP for reduction in pollutant loading ($/household)	
		Low end	High end	Low end	High end	Low end	High end
Greenhouse gases	771	750	779	774	769	772	770
Groundwater supply	27	33	22	24	29	27	27
Water purification	21	21	18	21	21	20	22
Total ecosystem services	819	819	819	819	819	819	819
Rice	0	0	0	0	0	0	0
Irrigated soybeans	121	98	152	125	119	129	118
Non-irrigated crop	543	502	582	410	650	528	555
Corn	1300	1221	1385	1343	1250	1305	1294
Cotton	54	120	0	56	50	55	52
CRP	1003	958	1093	998	1005	1001	1005
Total economic return	3021	2899	3212	2932	3074	3018	3024
Total of economic return and ecosystem service value (Value to society)	3840	3718	4031	3751	3893	3837	3843

The high-end value for the groundwater buffer value makes the groundwater supply value rise, but the water purification and greenhouse gas value fall to maintain the total ecosystem service value at Point D. The crop change associated with the new mix of ecosystem services include an increase in the non-irrigated crops and CRP, and a fall in the irrigated corn, soybeans, and cotton. The economic returns rise by 2% because a high-end buffer value for groundwater allows the total ecosystem service value requirement to be achieved more easily. When the low-end buffer value of groundwater is used, there is less non-irrigated crop, less CRP, and more irrigated crops, and the groundwater supply value declines and the greenhouse gas value rises. The low- and high-end WTP for pollutant loading reduction has minimal influence on the crops grown and the associated ecosystem service values. Less corn and more non-irrigated crop is grown with the high-end WTP value for water purification, and this slightly raises water purification value and slightly lowers greenhouse gas value. The economic returns are larger with the high-end value of the WTP, but this is an increase of less than 1%.

The hydro-conductivity of the aquifer measured at the high-end with greater lateral flow in the aquifer and shallower cones of depression or at the low-end with lower lateral flow in the aquifer and deeper cones of depression can also influence the crops grown to maximize economic returns (Table 5). The aquifer with high-end hydro-conductivity allows more irrigation intensive crops to be grown because the chance of aquifer exhaustion at some sites is lower. There is a rise in corn and a fall in non-irrigated crop while the other crops and the CRP are largely unchanged, and this increases economic returns by less than 1%. The associated ecosystem service change is that the groundwater supply value falls and the greenhouse gas value rises to maintain the required total value of ecosystem services at Point D. The low-end hydro-conductivity scenario indicates that corn declines and the non-irrigated crop rises because of the chance that groundwater pumping wells could go dry at some sites. The economic returns fall, and the total value of ecosystem service is maintained as greater groundwater supply value balances lower greenhouse gas value.

**Table 5:** Sensitivity of the present value of economic returns and ecosystem services to the hydro-conductivity of the aquifer and the pollutant loadings to surface water relative to point D on the efficiency frontier without reservoirs (in millions of 2015 constant dollars).

Ecosystem service or land cover	Point D on the efficiency frontier without reservoirs	Hydro-conductivity of the aquifer		Nutrient loading to surface water	
		Low-end	High-end	Low-end	High-end
Greenhouse gases	771	769	773	771	772
Groundwater supply	27	29	25	26	28
Water purification	21	21	21	22	19
Total ecosystem services	819	819	819	819	819
Rice	0	0	0	0	0
Irrigated soybeans	121	120	123	114	135
Non-irrigated crop	543	560	518	548	530
Corn	1300	1271	1341	1314	1289
Cotton	54	52	55	55	51
CRP	1003	1005	1001	998	1010
Total economic return	3021	3008	3038	3029	3015
Total of economic return and ecosystem service value (Value to society)	3840	3827	3857	3848	3834

The nutrient loadings to surface water lower the value of water purification, and this affects the crops grown because if crops generate greater nutrient loadings then less polluting crops must be grown to maintain the required total value of ecosystem services at Point D. High-end nutrient loading from the crops cause there to be less of the heavily polluting corn, cotton, and non-irrigated crops and more of the lightly polluting soybeans and CRP. Even though heavily polluting crops decline on the landscape, the high-end nutrient loading makes the water purification value fall, and the water supply and the greenhouse gas values rise. Economic returns fall with the high-end nutrient loading because less high value crops can be grown to maintain the required total value of ecosystem services. In the low-end nutrient loading scenario, more high economic value crops that are heavily polluting can be grown, and this means more corn, cotton, and non-irrigated crops. When all the crops are less polluting, the water purification value rises in spite of a shift toward the more heavily polluting crops, but the increases in irrigation intensive crops and the fall in CRP makes the water supply value and the GHG value fall.

Table 6 indicates the cost-share on reservoir construction cost increases the value to society from $2551 million to $2877 million (or 13%) because the water supply is larger and GHG emissions from fuel combustion fall. The tax on groundwater encourages a switch away from groundwater to reservoir water, rather than just an increase in reservoir water. The tax on groundwater has a lower economic cost per ecosystem dollar gained than the cost-share on reservoir construction.

**Table 6:** Present value of economic returns and ecosystem services that result when conservation policies influence the economic returns objective for the landscape with reservoirs (in millions of 2015 constant dollars).

Ecosystem service or land cover	Baseline (Point P)	Conservation policies			
		Cost-share reservoir construction costs ^a^	Tax on ground-water ^b^	Total maximum daily load ^c^	Carbon credits ^d^
Greenhouse gases	-2147	-1815	-1715	-1948	-1203
Groundwater supply	-56	-40	-39	-49	-20
Water purification	-2	-2	-2	7	-1
Total ecosystem services	-2206	-1857	-1755	-1991	-1224
Rice	932	962	902	963	754
Irrigated soybeans	698	716	687	670	690
Non-irrigated crop	587	523	597	482	644
Corn	2081	2083	2086	2056	2087
Cotton	439	437	442	373	445
CRP	20	12	25	111	50
Total economic return before government transfer	4757	4734	4738	4654	4669
Government transfer	0	98	-125	0	2521
Total of economic return before government transfer and ecosystem service value (Value to society)	2551	2877	2983	2663	3445
Economic cost per dollar of ecosystem service value gained (dollars) ^e^	--	0.07	0.04	0.48	0.09

^a^ The cost share for irrigation reservoir construction is 65% based on the rate from Natural Resource Conservation Service’s (USDA-NRCS) Agricultural Water Enhancement Program [22].

^b^ A tax on groundwater pumping cost of 15% is chosen to achieve groundwater conservation similar to the cost share on reservoir construction.

^c^ The total maximum annual load is chosen as the phosphorus and sediment exports from point L on the efficiency frontier with reservoirs.

^d^ The value of a carbon credit is $28.51 per metric ton of carbon according to the clearing price of the March 2015 auction by the European Union Emission Trading Scheme and an exchange rate of $0.87 per euro [23].

^e^ The economic cost per dollar of ecosystem service value gained is calculated as the difference in economic returns without and with the policy and dividing this by the difference in total ecosystem service value with and without the policy.

A total maximum daily load (TMDL) improves surface water quality by increasing land in rice, CRP, and reservoirs, and the water supply and GHG reduction value also increases. The increase in CRP land at the expense of corn makes the economic returns fall. The value to society from the TMDL rises only 4%, and the economic cost per ecosystem dollar gained is the highest of the policies. A carbon credit policy decreases rice and irrigated soybean, and the increases in reservoirs and sorghum reduce the GHG emissions from irrigation related fuel combustion. The carbon policy has a higher economic cost per ecosystem dollar gained than the cost-share on reservoir construction or the tax on groundwater.

## Conclusion

The use of efficiency frontiers to examine the tradeoff of economic returns and the value of ecosystem services indicate that a compromise among objectives generates more social value than directing the landscape exclusively to one objective. The social value of a landscape that incorporates economic and ecosystem service value is 30% greater than a landscape at the endpoint of a frontier where only ecosystem service or only economic value is taken into account. We find this compromise for the Arkansas Delta partly because corn generates strong economic returns and effectively sequesters GHG while using less irrigation water than rice. A higher social price for water purification makes polluting corn less effective at bridging economic and ecosystem objectives. Also many crops on the landscape, such as non-irrigated sorghum or irrigated soybeans, provide moderate economic returns without significantly harming ecosystem services.

Sensitivity analyses of the economic and ecosystem service model parameters reveal tradeoffs that occur among the land cover and ecosystem service values when the total value of ecosystem services is held at a constant level. The high-end social cost of carbon redirects the landscape toward more corn and CRP and less cotton, and this decreases groundwater supply value by 15% and water purification value by 20%. The high-end buffer value for groundwater or high-end WTP for a reduction in nutrient loadings directs the landscape toward more CRP and non-irrigated crops, and this lowers the GHG value by 1%. Higher prices for rice, soybeans, and corn decrease the groundwater supply value because all are irrigated crops while the GHG value increases for soybeans and corn by 1% and the water purification value increases for rice by 9%. The low-end hydro-conductivity reduces irrigated crops because the cones of depression deepen, and the greater groundwater conservation raises the groundwater supply value by 7.4% and the decline in corn lowers the GHG value. The high-end nutrient loadings to surface water decrease the corn and cotton and increase the CRP and soybeans, and the water purification value declines by 10%.

Reservoirs allow irrigated crop production to expand and use less groundwater, but the expansion of irrigated crops increase GHG emissions and surface water pollution. Reservoirs support a landscape with a higher value to society, up to 10% greater economic returns for a given level of all ecosystem services in some cases, but tradeoffs among ecosystem services mean that not necessarily all ecosystem services improve even when valued at their appropriate social value. Conservation policies can shift the landscape away from the maximum economic returns toward greater ecosystem service value, but this is done at an economic cost to society. Policies targeting groundwater conservation, either with a cost-share on reservoir construction costs or a tax on groundwater, increase the value of the ecosystem services at the least economic cost. The groundwater conservation policies are the most effective because this discourages GHG emissions from well pumping and discourages the production of the potent GHG emitting rice.

The methodology developed here is designed for use in an optimization model capable of creating efficiency frontiers for the use in long term planning by natural resource agencies when an agricultural landscape experiences groundwater overdraft. The usefulness of the efficiency frontier is that tradeoffs reflect the least that must be given up in ecosystem service value to obtain additional economic return. Given scarce budgets to achieve environmental and economic objectives, finding ways to achieve the most economic return with the least environmental degradation is worthwhile. However, assumptions of the economic and environmental components of the model are necessary for optimization to successfully occur and create efficiency frontiers. Significant assumptions of the economic model include constant prices for crops and ecosystem services and constant production costs for crops over time, although the consequence of these assumptions are explored with the sensitivity analyses. Significant assumptions on the ecosystem service model include the absence of interaction between surface water and groundwater except for a constant level of natural recharge to the aquifer and a lack of spatial interaction among the land covers chosen and the resulting surface water quality. As computing power and the solvers for optimization improve, these model additions can be included in analyses that use efficiency frontiers.

There is the potential for multiple lines of further inquiry for this research. The value of groundwater extends beyond its ability to augment surface water supplies and reduce the variability of the total water supply. Other values that relate to maintaining groundwater supply are the avoided damages from subsidence, prevention of saltwater intrusion, dilution of groundwater pollutants, and the support of terrestrial ecosystems through groundwater exfiltration and contributions to transpiration. Feedbacks between water conservation practice adoption such as reservoir construction and land prices is an issue not considered here, which could then drive cropping decisions on other agricultural land. Land market feedbacks and water conservation practice adoption factor into the discussion of policies to reduce groundwater overdraft [40, 41]. While we find that policies such as cost-share of reservoir construction or a total maximum daily load increase the social value of a landscape, we did not attempt to solve for the optimal level of the cost-share or limit on pollutant loadings that would maximize social net benefits. In addition to land-use change, a greater consideration of management practices, such as fertilizer application rates and tillage practices in agriculture, can provide additional options for performance.

## Supporting Information

S1 Supporting Information Five tables and one figure provide supplemental data, which expands upon value and model parameters discussed in this document. (DOCX)

## References

[1] Blomquist W, Heikkila T, Schlager E. 2001 Institutions and conjunctive water management among three western states. Nat Resour J. 41: 653–683.

[2] Noel J, Gardner BD, Moore C. Optimal Regional Conjunctive Water Management. Am J Agric Econ. 1980;62(3): 489–498.

[3] Popp J, Wailes E, Young K, Smartt J, Intarapapong W. Use of on-farm reservoirs in Rice production: results from the MARORA model. Journal of Agricultural and Applied Economics. 2003;35(2): 69–86.

[4] Arkansas Natural Resources Commission (ANRC). Arkansas water plan update 2014 | summary. Little Rock, AR; 2015.

[5] Chan KMA, Shaw MR, Cameron DR, Underwood EC, Daily GC. 2006 Conservation planning for ecosystem services. PLoS Biology 4:e379 doi: 10.1371/journal.pbio.0040379 17076586 10.1371/journal.pbio.0040379 PMC1629036

[6] Egoh B, Reyers B, Rouget M, Richardson DM, Le Maitre DC, van Jaarsveld AS. Mapping ecosystem services for planning and management. Agric Ecosyst Environ. 2008;127: 135–140.

[7] Egoh B, Reyers B, Rouget M, Bode M, Richardson DM. Spatial congruence between biodiversity and ecosystem services in South Africa.” Biol Conserv. 2009;142(3): 553–562. 10.1111/j.1523-1739.2009.01442.x 20136871

[8] Naidoo R, Balmford A, Costanza R, Fisher B, Green RE, Lehner B, et al Global mapping of ecosystem services and conservation priorities. Proceedings of the National Academy of Sciences. 2008;105: 9495–9500. 10.1073/pnas.0707823105 PMC2474481 18621701

[9] Raudsepp-Hearne C, Peterson G, Bennett E. Ecosystem service bundles for analyzing tradeoffs in diverse landscapes. Proceedings of the National Academy of Sciences. 2010;107: 5242–5247. 10.1073/pnas.0907284107 PMC2841950 20194739

[10] Kovacs K, Wailes E, West G, Popp J, Bektemirov K. Optimal spatial-dynamic management of groundwater conservation and surface water quality with on-farm reservoirs.” Journal of Agricultural and Applied Economics. 2014;46(4): 1–29.

[11] Nelson E, Polasky S, Lewis D, Plantinga A, Lonsdorf E, White D, et al Efficiency of incentives to jointly increase carbon sequestration and species conservation on a landscape. Proceedings of the National Academy of Sciences. 2008;105(28): 9471–9476. 10.1073/pnas.0706178105 PMC2474525 18621703

[12] Polasky S, Nelson E, Camm J, Csuti B, Fackler P, Lonsdorf E, et al Where to put things? spatial land management to sustain biodiversity and economic returns. Biol Conserv. 2008;141(6): 1505–1524.

[13] NOAA. National Climatic Data Center, climate at a glance, time series: 1895–2013. 2014. http://www.ncdc.noaa.gov/cag/.

[14] Vories ED, Evett SR. Irrigation research needs in the USDA mid-south and southeast, humid and sub-humid regions. Proceedings for the 5th National Decennial Irrigation Conference, ASABE #IRR10-8679, American Society of Agricultural and Biological Engineers (ASABE) and the Irrigation Association: Phoenix, AZ, M. Dukes (Ed.) 2010. pp. 1–12.

[15] Jenkins CT. Computation of rate and volume of stream depletion by wells: U.S. geological survey techniques of water-resources investigations. 4(D1); 17: 1968.

[16] Nalley L, Popp M, Fortin C. 2011 The impact of reducing greenhouse gas emissions in crop agriculture: a spatial and production level analysis. *Agricultural Resource Economics Review*. 2011;40: 63–80.

[17] Popp M, Nalley L, Fortin C, Smith A, Brye K. Estimating net carbon emissions and agricultural response to potential carbon offset policies. Agron J. 2011;103(4):1132–1143.

[18] Barker J, Baumgardner G, Turner D, Lee J. Potential carbon benefits of the Conservation Reserve Program in the United States. J Biogeogr. 1995; 22: 743–751.

[19] Tol RSJ. 2009 The economic effects of climate change. Journal of Economic Perspectives. 2009;23: 29–51.

[20] Tallis HT, Ricketts T, Guerry AD, Nelson E, Ennaanay D, Wolny S, et al. 2011. InVEST 2.1 beta user’s guide. the natural capital project. Stanford. 2011. http://www.naturalcapitalproject.org/InVEST.html. Cited 28 April 2013.

[21] Sharpley A. June 2013. Personal Communication. University of Arkansas.

[22] U.S. Department of Agriculture (USDA)—Natural Resources Conservation Service (NRCS), Arkansas. 2014 EQIP conservation practices and payment rates. http://www.nrcs.usda.gov/wps/portal/nrcs/detail/ar/home/?cid=STELPRDB1240703. Cited August 2014.

[23] European Commission Emission Trading Scheme. Auctions by the Transitional Common Auction Platform. 2015. http://ec.europa.eu/clima/policies/ets/cap/auctioning/docs/cap_report_201503_en.pdf. Cited July 2015.

[24] Flanders A, Baker R, Barber T, Faske T, Ginn H, Grimes C, et al. 2014 crop production budgets for farm planning. University of Arkansas Cooperative Extension Service, Division of Agriculture. 2014. http://www.uaex.edu/farm-ranch/economics-marketing/farm-planning/budgets/crop-budgets.aspx. Cited January 2015.

[25] Johnson DM, Mueller R. The 2009 cropland data layer. Photogramm Eng Remote Sensing. 2010: 1201–1205.

[26] U.S. Department of Agriculture (USDA)—National Agricultural Statistics Service (NASS) Arkansas Field Office. Soybean irrigated and non-irrigated. http://www.nass.usda.gov/Statistics_by_State/Arkansas/Publications/County_Estimates/. Cited November 2012.

[27] U.S. Department of the Treasury. Interest rate statistics. http://www.treasury.gov/resource-center/data-chart-center/interest-rates/Pages/default.aspx. Cited December 2012.

[28] Reed TB. Recalibration of a groundwater flow model of the Mississippi River Valley alluvial aquifer of Northeastern Arkansas, 1918–1998, with simulations of water levels caused by projected groundwater withdrawals through 2049." Little Rock, Arkansas: U.S. Geological Survey Water Resources Investigations Report. 2003: 03–4109.

[29] Barlow PM, Leake SA. Streamflow depletion by wells-understanding and managing the effects of groundwater pumping on streamflow. U.S. Geological Survey Circular 1376. 84: 2012.

[30] Clark BR, Westerman DA, Fugitt DT. Enhancements to the Mississippi Embayment Regional Aquifer Study (MERAS) groundwater-flow model and simulations of sustainable water-level scenarios. U.S. Geological Survey Scientific Investigations Report 2013–5161. 29: 2013.

[31] Tsur Y. The stabilization role of groundwater when surface water supplies are uncertain: the implications for groundwater development. Water Resources Research. 1990;26(5): 811–818.

[32] Kovacs K, Popp M, Brye K, West G. On-farm reservoir adoption in the presence of spatially explicit groundwater use and recharge. Journal of Agricultural and Resource Economics. 2015;40(1): 23–49.

[33] Great Pacific Trading Company (GPTC). Charts and quotes. http://www.gptc.com/quotes.html. Cited November 2015.

[34] U.S. Department of Agriculture (USDA)—Farm Service Agency (FSA). Conservation reserve program statistics. http://www.fsa.usda.gov/programs-and-services/conservation-programs/reports-and-statistics/conservation-reserve-program-statistics/index. Cited June 2015.

[35] Young KB, Wailes EJ, Popp JH, Smartt J. Value of water conservation improvements on Arkansas rice farms. *Journal of the ASFMRA*. 2004;67: 119–126.

[36] Smartt JH, Wailes EJ, Young KB, Popp JS. 2002. “MARORA (Modified Arkansas Off-Stream Reservoir Analysis) program description and user’s guide.” University of Arkansas. http://agribus.uark.edu/2893.php. Cited 28 April 2013.

[37] Arkansas Land Information Board. Five meter resolution digital elevation model. SDE Raster Digital Data. 2006. www.geostor.arkansas.gov/G6/Home.html?id=629c0f9562c2f9cd95ffd8ef564a5d7f. Cited May 2013.

[38] Hite D, Hudson D, and Intarapapong W. Willingness to pay for water quality improvements: the case of precision application technology. Journal of Agricultural and Resource Economics 2002;27(2): 433–449.

[39] Cole A. Arkansas Population Projections: 2003–2025. Center for Business and Economic Research, University of Arkansas, Fayetteville, 2003. http://cber.uark.edu/439.asp. Cited July 2013.

[40] Huffaker R, Whittlesey N. Agricultural water conservation legislation: will it save water? Choices 1995; 24–28.

[41] Ward FA, Pulido-Velazquez M. Water conservation in irrigation can increase water use.” Proceeding of the National Academy of Sciences. 2008;105 47: 18215–18220. 10.1073/pnas.0805554105 PMC2584147 19015510

