# Supplementary material for: The Influence of Groundwater Depletion from Irrigated Agriculture on the Tradeoffs between Ecosystem Services and Economic Returns
Source: PLoS One. 2016 Dec 29;11(12):e0168681. doi: 10.1371/journal.pone.0168681 (PMC5199064; doi:10.1371/journal.pone.0168681)
Supplement: S1 Supporting Information — (DOCX) [file pone.0168681.s001.docx]

**S1 Supporting Information**

Table A.1 indicates the initial 2014 crop mix and aquifer conditions for the study area. Well pumping reduces the size of the aquifer below the well and the aquifer of the surrounding sites. Water flows from the aquifer of surrounding sites into the site with the cone of depression created by the pumped well. The volume of the underground flow from surrounding sites depends on the distance from the pumped site and the hydraulic conductivity of the aquifer. Clark, Westerman and Fugitt (2013) use spatially coarse pilot points to digitize the hydraulic conductivity of the aquifer measured by feet per day. The groundwater model parameters are calibrated through a series of reports from the U.S. Geologic Survey (Clark, Westerman and Fugitt 2013; Gillip and Czarnecki 2009; Schrader 2010; Barlow and Leake 2012; Reed 2003). The spatial weight decides how much an acre-foot of water pumped from a well reduces the aquifer beneath the surrounding sites depends on the volume of underground flow.

**Table A.1.** Descriptive statistics of the spatially variable data across the sites of the study area

| **Variable** | **Definition** | **Mean** | **Std. Dev.** | **Sum**  **(thousands)** |
| --- | --- | --- | --- | --- |
| *L_i,rice_* , *L_i,corn_* , *L_i,cotton_* , *L_i,isoy_* , *L_i,dsoy_*  , *L_i,dsorg_*  , *L_i,dbl_* | Initial acres of rice, corn, cotton, irrigated soybean, dry land soybean, dry land sorghum, double crop irrigated soybean and winter wheat | 81; 52; 10; 165; 57; 7; 47 | 99; 77; 40; 97; 49; 23; 73 | 221; 143; 26; 449; 155; 20; 129 |
| *y_i,rice_* , *y_i,cotton_* , *y_i,corn_* , *y_i,isoy_* , *y_i,dsoy_* , *y_i,dsorg_* , *y_i,dbl_* , *y_i,wheat_* | Annual rice yield (cwt per acre), cotton yield (pounds per acre), corn, irrigated soybean, dry land soybean, dry land sorghum, double crop irrigated soybean, and winter wheat yields (bushels per acre) ^1^ | 71; 1054; 166; 42; 25; 66; 34; 57 | 3; 168; 11; 4; 3; 12; 1; 5 | - |
| *dp_i_* | Depth to water (feet) | 57 | 31 | - |
| *AQ_i_* | Initial aquifer size (acre-feet) | 27,587 | 12,514 | 82,016 |
| *K* | Hydraulic conductivity (feet per day) | 226 | 92 | - |
| *nr_i_* | Annual natural recharge of the aquifer per acre (acre-feet) | 0.001 | 0.04 | 547 |

Note: Number of sites is 2,724. ^1^ The mean and the standard deviation of the county yields come from the 11 counties in the study area.

Table A.2 indicates the economic and irrigation model parameters. Variable irrigation costs from wells or reservoirs include fuel, lube and oil, irrigation labor, and poly pipe for furrow irrigation plus the levee gates for the flood irrigation of rice (Hogan et al., 2007). The fuel cost per acre-foot of groundwater depends on the fuel needed to raise water from the aquifer, which depends on the depth to the aquifer and the efficiency of the pump, gear-head and motor. The groundwater pump is assumed to deliver 1,800 gallons per minute and the stationary re-lift pump for the reservoir and tail-water recovery system to deliver 2,000 gallons per minute (Hogan et al., 2007). The range in diesel use for a well is 13 gallons of diesel per acre foot for a 100 foot well to 26 gallons of diesel per acre foot for a 200 foot well, and 6 gallons of diesel is needed per acre-foot for pumping water to and from the reservoir (Division of Agriculture, 2012). We add 10% to the fuel cost of $3.77 per gallon of diesel fuel (EIA, 2012) to account for the oil and lube for irrigation equipment (Hogan et al., 2007). The capital cost of the irrigation equipment is amortized and divided by the acre-feet pumped from the well to calculate a capital cost per acre-foot pumped (Hogan et al., 2007).

On-farm reservoir/tail-water recovery construction and maintenance costs for various reservoir sizes were estimated using Modified Arkansas Off-Stream Reservoir Analysis (MARORA) to obtain capital cost estimates (Smartt et al., 2002). Since a majority of the construction cost for a reservoir rests on the cost to move one cubic yard of soil, this cost was updated from $1 per cubic yard to $1.2 per cubic yard to reflect changes in fuel cost since 2002. The remainder of the investment and maintenance cost comes from MARORA estimates and includes re-lift pumps for moving water from the tail-water pit to the reservoir and from the reservoir to the field. The reservoir and tail-water recovery system capital cost is amortized to annual per acre payments. While reservoirs are currently in use, we do not have information on their location, and thus assume reservoirs occupy no land in the initial period of the model.

**Table A.2.** Value of economic and irrigation model parameters.

| **Parameter** | **Definition** | **Value** |
| --- | --- | --- |
| *pr_rice_* , *pr_cot_* , *pr_corn_* , *pr_soy_* , *pr_sorg_* , *pr_wht_* , *pr_crp_* | Price of rice ($/cwt), cotton ($/lbs), corn, soybeans, sorghum, and wheat ($/bushel), and the government payment per acre for CRP | 14.00, 0.88, 5.50, 11.99, 5.23, 6.39, 69.5 |
| *ca_rice_* , *ca_corn_* , *ca_cotton_* , *ca_isoy_* , *ca_dsoy_*  , *ca_dsorg_*  , *ca_dbl_* , *ca_wht_* , *ca_crp_* | Annual production cost excluding irrigation fuel for rice, corn, cotton, irrigated soybean, non-irrigated soybean, non-irrigated sorghum, double crop irrigated soybean, winter wheat, and conservation reserve program ($/acre) | 646, 605, 715, 326, 289, 270, 326, 307, 26 |
| *wd_rice_* , *wd_corn_* , *wd_cotton_* , *wd_isoy_* , *wd_dbl_* | Annual irrigation per acre of rice, corn, cotton, full-season soybean, and double crop soybean (acre-feet) | 2.5, 1.0, 1.0, 1.0, 0.75 |
| $\omega_{\min}$,$\omega_{\max}$ | Annual minimum and maximum capacity of a one acre reservoir (acre-feet) | 1.4, 11 |
|  | Estimated annual per acre cost of reservoir ($/acre) | 376.8^a^ |
|  | Cost to re-lift an acre-foot to and from the reservoir ($/acre-foot) | 22.62 |
| *c^p^* | Cost to raise an acre-foot of water by one foot ($/foot) | 0.55 |
| $\delta_{t}$ | Discount factor | 0.95 |
| $\xi_{i}$, | Soil factor, fraction of carbon lost to respiration due to soil related microbial activity | 0.72 |

^a^ This is the amortized cost to construct an additional acre of reservoir. The first acre of the reservoir constructed is more expensive, and the last acre of reservoir constructed is less expensive.

The next two tables indicate the water purification model parameters for nutrient and sediment pollution, respectively. Table A.3 shows the values for nutrient loading, evapotranspiration, rooting depth, available water capacity, and vegetation filtering. Table A.4 reports the values for crop/vegetation and management factor, support practice factor, and sediment filtering. We have calibrated the water purification models with the following reports and publications (Allen et al., 1998; Dwyer et al., 1998; Manley et al., 2009; Mishra et al., 1997; Moore et al., 1993; Phocaides, 2007; Reckhow et al., 1980; Reddy, 1982; Simpson et al., 2008; Tallis et al., 2011; USDA-NRCS, 2004; Wischmeier & Smith, 1978; Zaines & Schultz, 2002; USDA, 2012).

The water yield is the difference between precipitation and actual evapotranspiration of each site. Digital maps for these data include the 30-year mean annual precipitation (Prism Climate Group, 2010), while the actual evapotranspiration depends on the potential evapotranspiration (Ahn and Tateishi, 1994), soil depth and plant available water content (USDA-NRCS, 2013), and rooting depth (Schenk and Jackson, 2002) by land cover (Table A.3).

The quantity of nutrients leaving each site depends on the export coefficients and filtering characteristics of each land cover (Table A.3), the water yield from before, and the slope of the land from a digital elevation model (Arkansas Land Information Board, 2006). The sediment transport to a stream follows a universal soil loss equation (Wischmeier and Smith, 1978) which uses rainfall erosivity (EPA, 2013), soil erodibility (USDA-NRCS, 2013), slope-length gradient factor (Arkansas Land Information Board, 2006), crop management factor (e.g. tillage), and support practices (e.g. cross slope versus downslope furrows). The crop management and support practice factors as well as the sediment filtering depend on the land cover (Table A.4).

**Table A.3.** Values for nutrient loading, evapotranspiration, rooting depth, available water capacity, and vegetation filtering.

| **LULC** | **Evapotranspiration** | **Rooting**  **depth** | **Phosphorus**  **loading** | **Phosphorus**  **filtering** | **Nitrogen**  **loading** | **Nitrogen**  **filtering** |
| --- | --- | --- | --- | --- | --- | --- |
| Corn | 1200(e) | 900(c) | 2210(a) | 25(b) | 12420(a) | 50(d) |
| Cotton | 1200(e) | 1000(j) | 4310(a) | 25(b) | 9310(a) | 25(b) |
| Rice | 1200(e) | 550 (i) | 450(f) | 80(h) | 600(f) | 90(l) |
| Soybeans, Dbl Crop Winter Wht/Soybean | 1150(e) | 740(c) | 1907(k) | 62(k) | 4712(k) | 70(k) |
| Sorghum, Sunflower, Winter Wheat, Oats, Millet, Safflower, Other Crops, Peas, Peaches, Pecans, Squash, Dbl Crop Winter Wht/Corn, Dbl Crop Soybeans/Oats, Cabbage | 600(b) | 700(b) | 2320(a) | 62(k) | 5630(a) | 70(k) |
| Fallow/Idle Cropland | 200(b) | 500(b) | 100(b) | 50(b) | 3400(b) | 50(b) |
| Pasture/Hay | 850(b) | 1000(b) | 100(b) | 25(b) | 3100(b) | 25(b) |
| Open Water | 1000(b) | 1000(b) | 1(b) | 5(b) | 1(b) | 5(b) |
| Developed/Open Space, Developed/Low Density, Developed/Medium Density, Developed/High Density | 100(b) | 10(b) | 500(b) | 5(b) | 4000(b) | 5(b) |
| Barren | 200(b) | 10(b) | 1(b) | 5(b) | 4000(b) | 5(b) |
| Deciduous Forest, Evergreen Forest, Mixed Forest, Shrubland | 1000(b) | 7000(b) | 35(a) | 70(g) | 2862(a) | 80(b) |
| Grassland Herbaceous | 650(b) | 2000(b) | 50(b) | 60(g) | 4000(b) | 40(b) |
| Woody Wetlands, Wetlands | 1000(b) | 7000(b) | 50(b) | 80(b) | 2000(b) | 80(b) |

Source: a) Reckhow et al., 1980; b) Tallis et al., 2011; c) Dwyer et al., 1998; d) Simpson et al., 2008; e) Allen et al., 1998; f) Manley et al., 2009; g) Zaines & Schultz, 2002; h) Moore et al., 1993; i) Mishra et al., 1997; j) Phocaides, 2007; k) USDA, 2012; l) Reddy, 1982.

**Table A.4.** Values for crop/vegetation and management factor, support practice factor, and sediment filtering.

| LULC | Crop/vegetation and management factor | Support practice factor | Sediment filtering |
| --- | --- | --- | --- |
| Corn | 130(c) | 400(c) | 25(a) |
| Cotton | 170(c) | 400(c) | 25(a) |
| Rice | 90(c) | 400(c) | 25(a) |
| Soybeans, Dbl Crop Winter Wht/Soybean | 120(c) | 400(c) | 25(a) |
| Sorghum, Sunflower, Winter Wheat, Oats, Millet, Safflower, Other Crops, Peas, Peaches, Pecans, Squash, Dbl Crop Winter Wht/Corn, Dbl Crop Soybeans/Oats, Cabbage | 170(c) | 400(c) | 25(a) |
| Fallow/Idle Cropland | 8(c) | 200(c) | 5(a) |
| Pasture/Hay | 20(a) | 250(a) | 40(a) |
| Open Water | 1(a) | 1(a) | 80(a) |
| Developed/Open Space, Developed/Low Density, Developed/Medium Density, Developed/High Density | 1(a) | 1(a) | 5(a) |
| Barren | 250(a) | 10(a) | 20(a) |
| Deciduous Forest, Evergreen Forest, Mixed Forest, Shrubland | 3(b) | 200(b) | 60(a) |
| Grassland Herbaceous | 8(c) | 200(c) | 40(a) |
| Woody Wetlands, Herbaceous Wetlands | 10(a) | 200(a) | 60(a) |

Source: a) Tallis et al., 2011; b) Wischmeier & Smith, 1978; c) USDA-NRCS, 2004

The carbon sequestered by aboveground biomass per acre (Eq. A.1) for crop $j$ in site $i$ ($AGB_{ij}$) is

,

(A.1)

where $Y_{ij}$ is the grain or fiber yields in conventional units per acre for crop $j$ in site $i$, and yield is converted to tons per acre using $\lambda_{j}$, and then to dry mass using the moisture content for the (wet) yield of the crop *j* with $\alpha_{j}$. The harvest index, $H_{j}$, uses the crop yield to determine the aboveground biomass such as stems and leaves that remain on the field after harvest. The harvested grain or fiber once beyond the farm gate does not affect GHG reduction although products such as clothing from cotton can store carbon as effectively as soil. To convert the above ground biomass into tons of carbon sequestrated, the proportion of plant residue incorporated in the soil depends on tillage methods for crop j, $\delta_{j}$, and tillage affects the fraction, $\eta_{j}$, of carbon from incorporated plant residue remaining in the soil after microbial decomposition. The estimated carbon concentration of aboveground biomass is $\beta_{j}$.

The carbon sequestrated from the belowground biomass (Eq. A.2) per acre for crop $j$ in site $i$ ($BGB_{ij}$) is estimated by

,

(A.2)

where, like in equation (8), the dry mass of the yield in tons per acre is determined with $Y_{ij}$, $\lambda_{j}$, and $\alpha_{j}$. The shoot/root ratio $\varphi_{j}$ divided by the harvest index $H_{j}$ converts the yield to belowground biomass of which only a fraction, $\eta_{j}$, with tillage affecting microbial decomposition of incorporated plant residue with an estimated carbon concentration of $\chi_{j}$.

Carbon sequestration from both AGB and BGB is further discounted to reflect differences in carbon cycling due to soil texture as described above. The area average soil texture effect was 0.72 owing to differences in carbon retention at 1.0 for clayey soils, 0.7 for loamy soils and 0.4 for sandy soils as in Popp et al. (2011). That is, only 72% of the carbon sequestered in the soil due to root or plant tissue decay remains in the soil given soil dynamics. Table A.5 summarizes the carbon model parameters used while Figure A.1 providing a visual summary of GHG emission and sequestration differences across crops and CRP.

**Table A.5.** Value for carbon model parameters.

| **Parameter** | **Definition** | **Value** |
| --- | --- | --- |
| $\lambda_{rice}$,$\lambda_{corn}$,$\lambda_{cotton}$,$\lambda_{isoy}$,$\lambda_{dsoy}$,  $\lambda_{dsorg}$,$\lambda_{dbl}$, $\lambda_{wht}$ | Yield multiplier to convert from conventional yield units to kg per acre for rice (hundred weight), corn (bushels), cotton (pounds of lint), irrigated soybean (bushels), non-irrigated soybean (bushels), non-irrigated sorghum (bushels), double crop irrigated soybean (bushels), and wheat (bushels) | 45.5, 25.4, 1.19, 27.2, 27.2, 25, 27.2 |
| $\alpha_{rice}$, $\alpha_{corn}$,$\alpha_{cotton}$,$\alpha_{isoy}$,  $\alpha_{dsoy}$, $\alpha_{dsorg}$, $\alpha_{dbl}$, $\alpha_{wht}$ | Moisture content (wet basis) of rice, corn, cotton, irrigated soybean, non-irrigated soybean, non-irrigated sorghum, and double crop irrigated soybean and winter wheat | 0.13, 0.155, 0, 0.13, 0.13, 0.14, 0.13, 0.135 |
| $H_{rice}$, $H_{corn}$, $H_{cotton}$, $H_{isoy}$, $H_{dsoy}$, $H_{dsorg}$, $H_{dbl}$, $H_{wht}$ | Harvest index (grain weight to total above ground biomass weight) of rice, corn, cotton, irrigated soybean, non-irrigated soybean, non-irrigated sorghum, and double crop irrigated soybean and winter wheat | 0.45, 0.43, 0.45, 0.45, 0.45, 0.39,  0.45, 0.46 |
| $\beta_{rice}$, $\beta_{corn}$, $\beta_{cotton}$, $\beta_{isoy}$, $\beta_{dsoy}$, $\beta_{dsorg}$,$\beta_{dbl}$,$\beta_{wheat}$ | Crop residue C content of rice, corn, cotton, irrigated soybean, non-irrigated soybean, non-irrigated sorghum, and double crop irrigated soybean and winter wheat in g per kg. | 360, 410, 420, 430, 430, 420, 430, 340 |
| $\delta_{low}$ , $\delta_{conventioal}$ | Fraction of aboveground biomass C remaining in the soil with low tillage, and conventional tillage | 0.40, 0.70 |
| $\eta_{low}$ , $\eta_{conventional}$ | Fraction of belowground biomass C remaining in the soil with low tillage, and conventional tillage | 0.45, 0.40 |
| $\chi_{rice}$, $\chi_{corn}$,$\chi_{cotton}$,$\chi_{isoy}$,  $\chi_{dsoy}$,$\chi_{dsorg}$, $\chi_{dbl}$,$\chi_{wheat}$ | Root C content of rice, corn, cotton, irrigated soybean, non-irrigated soybean, non-irrigated sorghum, and double crop irrigated soybean and winter wheat in g per kg. | 350, 420, 360, 430, 430, 380, 430, 280 |
| $\phi_{rice}$,$\phi_{corn}$,$\phi_{cotton}$,$\phi_{isoy}$,  $\phi_{dsoy}$,$\phi_{dsorg}$,$\phi_{dbl}, \phi_{wheat}$ | Root/shoot ratio (below ground biomass weight / above ground biomass weight) of rice, corn, cotton, irrigated soybean, non-irrigated soybean, non-irrigated sorghum, and double crop irrigated soybean and winter wheat | 0.16, 0.19, 0.21, 0.16, 0.16, 0.08, 0.16, 0.18 |
| $\sigma_{g}$, $\sigma_{r}$ | Conversion factors to track the carbon emitted from fuel combustion to lift an acre-foot of water one foot and the carbon emitted from fuel combustion to pump an acre-foot of water into a reservoir and back out to the field. | 10.37, 190.95 |

Source: Popp et al. (2011)

**Figure A.1.** Summary of Soil Carbon Sequestration and GHG Emissions by Crop.

Note: Error bars provide the range of sequestration and irrigation fuel use emissions associated with yield and irrigation depth and irrigation type (well vs reservoir) changes across the study region. Additional variation is modeled with changes in soil texture but not included in this diagram. Conservation reserve program carbon equivalent footprint is taken from Barker et al. (1995) where regional variation due to yield is not taken into consideration.

**Appendix references**

Ahn, C.H., and R. Tateishi. 1994. “Development of a Global 30-minute grid Potential Evapotranspiration Data Set.” *Journal of the Japan Soc. Photogrammetry and Remote Sensing*, 33(2):12-21.

Barker, J., G. Baumgardner, D. Turner, and J. Lee. 1995. “Potential carbon benefits of the Conservation Reserve Program in the United States.” *Journal of Biogeography* 22: 743-751.

Barlow, P.M., S.A. Leake. “Streamflow Depletion by Wells-Understanding and Managing the Effects of Groundwater Pumping on Streamflow.” U.S. Geological Survey Circular 1376, 84 p. 2012.

Allen, R.G., L.S. Pereira, D. Raes, and M. Smith. 1998. “Crop evapotranspiration. Guidelines for computing crop water requirements.” *FAO Irrigation and Drainage Paper 56, Rome*. Internet site: <http://www.fao.org/docrep/x0490e/x0490e0b.htm> (Accessed June 28, 2013)

Arkansas Land Information Board. *2006.* “Five Meter Resolution Digital Elevation Model*.*” *SDE Raster Digital Data*. Internet site: <http://www.geostor.arkansas.gov/G6/Home.html?id=629c0f9562c2f9cd95ffd8ef564a5d7f> (Accessed May, 2013)

Clark, B.R., D.A. Westerman, and D.T. Fugitt. 2013. “Enhancements to the Mississippi Embayment Regional Aquifer Study (MERAS) groundwater-flow model and simulations of sustainable water-level scenarios*.*” *Reston, Virginia: U.S. Geological Survey Scientific Investigations Report*: 2013–5161.

Division of Agriculture - University of Arkansas. 2013. “2014 Crop Enterprise Budgets.” Little Rock, AR: AG-1292. Internet site: <http://www.uaex.edu/farm-ranch/economics-marketing/docs/Budgets%202014.pdf>

Dwyer, L.M., D.W. Stewart, and D. Balchin. 1998. “Rooting Characteristics of Corn, Soybeans and Barley as a Function of Available Water and Soil Physical Characteristics.” *Canadian Journal of Soil Science.* 68: 121-132

Energy Information Administration (EIA) - U.S. Department of Energy. “Gasoline and Diesel Fuel Update.” Internet site: <http://www.eia.gov/petroleum/gasdiesel/> (Accessed November 2012)

Environmental Protection Agency. 2013 “Storm Water Phase II Final Rule.” *Washington, DV: US EPA publication Fact Sheet 3.1*. Internet site: http://www.epa.gov/esd/land-sci/ (Accessed May, 2013)

Gillip, J.A., and Czarnecki, J.B., 2009, Validation of a ground-water flow model of the Mississippi River Valley alluvial aquifer using water-level and water-use data for 1998-2005 and evaluation of water-use scenarios: U.S. Geological Survey Scientific Investigations Report 2009-5040, 22 p.

Hogan, R., S. Stiles, P. Tacker, E. Vories, and K. J. Bryant. 2007. “Estimating irrigation costs.” Little Rock, AR: Arkansas Cooperative Extension Service. FSA28-PD-6-07RV.

Manley, S.W., R.M. Kaminski, P.B. Rodrigue, J.C. Dewey, S.H. Schoenholtz, P.D. Gerard, and K.J. Reinecke. 2009. “Soil and nutrient retention in winter-flooded ricefields with implications for watershed management.” *Journal of Soil and Water Conservation* 64,(3): 173-182

Mishra, H.S., T.R. Rathore, and R.C. Pant. 1997. “Root growth, water potential, and yield of irrigated rice.” *Irrigation Science* 17: 69–75

Moore, P.A., K.K. Baugh, R.J. Norman, B.R. Wells, and R.S. Helms. “Nutrient runoff from rice fields.” *Arkansas Rice Research Studies 1992*. B.R. Wells, ed. University of Arkansas Agricultural Experiment Station Research Series 431(1993): 142-149.

Phocaides, A. *Handbook on Pressurized Irrigation Techniques*. Second Edition. Rome, Italy: Food and Agriculture Organization of the United Nations, 2007. Internet site: <ftp://ftp.fao.org/docrep/fao/010/a1336e/a1336e06.pdf> (Accessed May, 2013)

Popp, M., L. Nalley, C. Fortin, A. Smith, and K. Brye. 2011. “Estimating Net Carbon Emissions and Agricultural Response to Potential Carbon Offset Policies.” *Agronomy Journal* 103(4):1132-1143.

Prism Climate Group. “30-year Normal Precipitation: Annual (mm*100).” Oregon State University (October 2010). Internet site: <http://www.prism.oregonstate.edu/>. Updated Monthly.

Reckhow, K.H., M.N. Beaulac, and J.T. Simpson. 1980. “Modeling phosphorus loading and lake response under uncertainty: a manual and compilation of export coefficients”*.* Washington, DC: USA EPA/440/5-80/011.

Reed, T.B. 2003. “Recalibration of a Groundwater Flow Model of the Mississippi River Valley Alluvial Aquifer of Northeastern Arkansas, 1918-1998, with Simulations of Water Levels caused by Projected Groundwater Withdrawals Through 2049." Little Rock, Arkansas: U.S. Geological Survey Water Resources Investigations Report 03-4109.

Reddy, K.R. 1982. “Nitrogen cycling in a flooded-soil ecosystem plated to rice (*Oryza sativa L*).” *Plant and Soil* 67: 209-220.

Schenk, H.J., and R.B. Jackson. 2002. “The Global Biogeography of Roots.” *Ecological Monographs* 72(3): 311-328.

Simpson, T.W., A.N. Sharpley, R.W. Howarth, H.W. Paerl, and K.R. Mankin. 2008. “The New Gold Rush: Fueling Ethanol Production while Protecting Water Quality.” *Journal of Environmental Quality* 37: 318–324.

Smartt, J.H., E.J. Wailes, K.B. Young, and J.S. Popp. 2002. “MARORA (Modified Arkansas Off-Stream Reservoir Analysis) Program Description and User’s Guide.” Unpublished manuscript. University of Arkansas. Internet site: <http://agribus.uark.edu/2893.php> (Accessed April 28, 2013).

Tallis, H.T., T. Ricketts, A.D. Guerry, E. Nelson, D. Ennaanay, S. Wolny, N. Olwero, K. Vigerstol, D. Pennington, G. Mendoza, J. Aukema, J. Foster, J. Forrest, D. Cameron, E. Lonsdorf, C. Kennedy, G. Verutes, C.K. Kim, G. Guannel, M. Papenfus, J. Toft, M. Marsik, J. Bernhardt, S.A. Wood, and R. Sharp. *InVEST 2.1 beta User’s Guide.* The Natural Capital Project, Stanford, 2011. Internet site: <http://www.naturalcapitalproject.org/InVEST.html> (Accessed April 28, 2013).

USDA. 2012. “Assessment of the Effects of Conservation Practices on Cultivated Cropland in the Upper Mississippi River Basin.” *Revised CEAP Report*: 57-65.

----------. NRCS. “Arkansas RUSLE Cropping Management Regions.” Agronomy Technical Note No. 2. Technical Guide Reference No. January 2, 2004.

----------. NRCS. Soil Survey Staff, *Soil Survey Geographic (SSURGO) Database for Arkansas*, 2013. Internet site: <http://soildatamart.nrcs.usda.gov> (Accessed April 28, 2013).

Wischmeier, W.H., and D. Smith. 1978 “Predicting rainfall erosion losses: a guide to conservation planning.” Washington, DC: USDA-ARS Agriculture Handbook.

Zaines, G.N., and R.C. Schultz. 2002. “Phosphorus in Agricultural Watersheds. A Literature Review.” *Department of Forestry, Iowa State University, Ames, Iowa*.
